# IOTTRUST: graph-based anomaly detection for IoT intrusion using network flow topology and community structure analysis on UNSW-NB15

**DOI:** 10.3389/fdata.2026.1885965

**Published:** 2026-09-10

**Authors:** Nachaat Mohamed, Hamed Taherdoost

**Affiliations:** 1 Homeland Security Department, Rabdan Academy, Abu Dhabi, United Arab Emirates; 2 NEUSAI Technologies LLC, Sharjah, United Arab Emirates; 3 Department of Arts, Communications and Social Sciences, School of Arts, Science and Technology, University Canada West, Vancouver, BC, Canada; 4 GUS Institute, Global University Systems, London, United Kingdom; 5 College of Technology and Engineering, Westcliff University, Irvine, CA, United States; 6 Research and Development Department, Hamta Business Corporation, Vancouver, BC, Canada; 7 Faculty of Information Technology, Victorian Institute of Technology, Melbourne, VIC, Australia

**Keywords:** anomaly detection, community detection, degree centrality, false positive reduction, fan-out analysis, feature engineering, graph-based IDS, IoT security

## Abstract

**Introduction:**

Conventional machine learning approaches to IoT intrusion detection treat each network flow record as an independent observation, discarding the relational structure that connects flows across source IPs, destination IPs, and subnet communities. This article presents IOTTRUST, a graph-augmented intrusion detection framework for IoT networks that constructs a directed network flow graph from the UNSW-NB15 dataset and enriches per-flow machine learning with 24 graph-derived topology features computed per source and destination IP.

**Methods:**

All graph topology statistics are computed exclusively from the training partition and propagated to the test partition without access to test-set labels, eliminating the temporal leakage present in an earlier version of this study. The experimental design isolates the contribution of graph topology through a three-track comparison conducted on an identical 40,000-flow stratified sample with a fixed 70/30 split (random seed 42): a Flow-Only baseline using the standard 43 UNSW-NB15 features, a Graph-Only model trained exclusively on 24 graph topology features, and the full IOTTRUST Hybrid model combining 22 numeric flow features with 24 graph features (46 total).

**Results:**

Under this leakage-free, size-matched evaluation, Flow-Only achieves 98.98% accuracy and AUC = 0.9995 with FPR = 0.81%; Graph-Only alone reaches 97.89% accuracy and AUC = 0.9974 with FPR = 1.79%; and IOTTRUST Hybrid achieves 99.07% accuracy, AUC = 0.9996, Precision = 97.09%, Recall = 98.30%, and FPR = 0.74%, a modest but statistically significant improvement over Flow-Only (McNemar *p* = 0.295 on the held-out test set, paired *t*-test on five-fold cross-validated *F*1 *p* = 0.026) and a substantial improvement over Graph-Only alone (McNemar *p* < 0.001).

**Discussion:**

Community structure analysis on the training graph confirms that the attacker subnet (175.45.176.x, four IPs) accounts for over 90% of attack flows, exhibiting distinctive graph signatures that graph features capture directly. Feature importance analysis shows that 12 of the top 15 most important features in the leakage-free Hybrid model remain graph topology features, accounting for 60.4% of the top-15 importance mass, confirming that network relational structure continues to carry predictive information even under this stricter evaluation protocol.

## 1 Introduction

The proliferation of Internet of Things (IoT) devices has fundamentally transformed the attack surface of modern networks. By 2025, an estimated 30 billion IoT devices were actively connected globally (Statista, 2024), spanning industrial control systems, smart home infrastructure, medical devices, and urban sensing networks. Each device category introduces distinctive traffic patterns and distinct vulnerability surfaces; collectively, they generate the high-volume, heterogeneous network traffic that fundamentally challenges traditional signature-based intrusion detection systems. Machine learning-based IDS approaches have emerged as the dominant research response to this challenge, achieving impressive accuracy figures on standard benchmarks such as UNSW-NB15 (Moustafa and Slay, 2015), CICIDS-2017, and TON-IoT (Moustafa, 2021). Yet virtually all of these approaches share a design decision that, on close examination, is difficult to justify: they treat each network flow as a statistically independent observation.

The independence assumption carries a clear computational rationale: it reduces intrusion detection to a standard supervised classification problem amenable to well-understood algorithms and evaluation protocols. However, it fundamentally misrepresents the relational structure of IoT network attacks. When a botnet launches a reconnaissance sweep across an IoT subnet, it is not generating 10,000 independent flows; it is generating a coordinated, directional pattern in which a small set of source IPs fan out to a large number of destination IPs in a short time window (Luo et al., 2024). When a single attacker IP conducts SQL injection attempts against a web server, the resulting flows share a source-destination pair and a protocol signature that creates detectable clustering in the network flow graph. These graph-level signatures high out-degree for attacker nodes, elevated fan-out ratios, anomalous betweenness centrality for pivot nodes, and tight community structure in attacker subnets are invisible to any method that processes flows one at a time (Lo et al., 2022).

Graph-based approaches to network security have a well-established theoretical foundation. Intrusion detection graphs were proposed as early as 2002 by Ning et al. (2002) in the context of multi-step attack reconstruction, and network flow graphs have been used for malware detection (Zhao et al., 2022), botnet identification (Zhao et al., 2023), and traffic anomaly detection (Peng et al., 2023). However, the application of graph topology features as an *enrichment layer* for per-flow machine learning classifiers rather than as a replacement has received limited systematic attention on contemporary IoT benchmark datasets. The UNSW-NB15 dataset, despite its widespread use, has never been analyzed from the perspective of its network flow graph structure: the 47 unique IP addresses that appear in the dataset, their connection patterns, their community membership, and the topological properties that distinguish attacker IPs from victim and server IPs. IOTTRUST addresses this gap comprehensively.

The contributions of this article are as follows:

1. We construct, to the best of our knowledge, the first directed network flow graph from the full UNSW-NB15 dataset (440,047 flow edges, 47 IP nodes, 10 subnet communities) and conduct a systematic community structure analysis, revealing that the attacker subnet 175.45.176.x (four IPs) accounts for 89.85% of all attack flows in the full dataset and 100% of attack flows within the training partition used for graph construction (community attack ratio = 0.90), and exhibits graph signatures fan-out ratio up to 0.2553, attack_ratio_src up to 0.9632 that are unambiguously separable from normal-traffic nodes.
2. We design and evaluate three complementary detection models Flow-Only (43 or 22 features, depending on the analysis), Graph-Only (24 features), and IOTTRUST Hybrid (46 features) on an identical 40,000-flow leakage-free sample with a fixed 70/30 split, demonstrating that the Hybrid model achieves the best overall performance (99.07% accuracy, AUC = 0.9996, FPR = 0.74%) while Graph-Only alone (97.89% accuracy) trails the full-feature Flow-Only baseline (98.98% accuracy) under this stricter, size-matched protocol.
3. We demonstrate that, under a leakage-free and size-matched evaluation, graph feature enrichment reduces the IOTTRUST Hybrid model's false positive rate to 0.74%, a reduction of 9.0% relative to the strong Flow-Only baseline (FPR = 0.81%) and 58.7% relative to Graph-Only alone (FPR = 1.79%), with the improvement over Flow-Only confirmed as statistically significant by a paired *t*-test on five-fold cross-validated *F*1-scores (*p* = 0.026), a result of direct operational significance in IoT environments where false positive fatigue is a primary driver of IDS alert suppression.
4. We perform systematic feature importance analysis showing that 12 of the top 15 leakage-free IOTTRUST Hybrid features are graph topology features, with g_dst_AR_dst (0.1544), sttl (0.1146), and g_src_AR_src (0.1144) being the three most important features overall, together accounting for 38.3% of the top-15 importance mass providing interpretable security insights beyond classification accuracy.
5. We validate the statistical significance of all comparative claims using McNemar's test on held-out test-set predictions and paired *t*-tests on five-fold cross-validated *F*1-scores, quantify the computational overhead of graph construction and per-flow feature extraction, and position IOTTRUST against recent GraphSAGE-based intrusion detection systems evaluated on UNSW-NB15, establishing that lightweight graph statistics deliver competitive detection performance without the training overhead of a full graph neural network pipeline.

What distinguishes IOTTRUST from prior work is a combination of factors that, to the best of our knowledge, has not been jointly addressed in the existing literature. First, while GNN-based approaches such as E-GraphSAGE (Lo et al., 2022) also exploit relational structure, they require a full end-to-end graph neural network training pipeline and offer limited interpretability; IOTTRUST, by contrast, uses lightweight graph statistics as an enrichment layer atop a conventional Random Forest, delivering comparable accuracy with transparent, operationally meaningful features and without GPU-based training. Concretely, E-GraphSAGE reports 96.8% binary accuracy on UNSW-NB15 (Lo et al., 2022), and a more recent SAGEConv-Transformer hybrid reports 98.41% accuracy and a macro-*F*1 of 0.9749 on the same dataset using a five-seed GNN training regime (Zhang et al., 2026). It must be noted, however, that these figures are drawn from the respective original publications, each conducted under evaluation protocols that differ from the leakage-free, size-matched 40,000-flow protocol adopted in this article; accordingly, the numerical comparison is indicative of relative performance and training cost rather than a strictly controlled head-to-head benchmark, and a controlled re-implementation of E-GraphSAGE under the identical leakage-free protocol is identified as a priority direction for future work (Section 8). Within these caveats, the leakage-free IOTTRUST Hybrid model (99.07% accuracy, *F*1 = 97.69%) is competitive with both of these GNN-based systems while requiring only graph statistics that can be computed in O(|F|) time with no neural network training, making it substantially more accessible for deployment on resource-constrained IoT gateways. Second, unlike prior work that uses NetFlow-level graphs for botnet detection (Zhao et al., 2023; Ding et al., 2023) or encrypted traffic classification (Zhou et al., 2022), IOTTRUST constructs a directed, weighted network flow graph from a standard IoT intrusion detection benchmark (UNSW-NB15) and systematically quantifies the contribution of graph topology through a controlled, leakage-free three-track experimental design. Third, the modest but statistically significant FPR reduction demonstrated by IOTTRUST under the leakage-free protocol directly addresses the practical operational challenge of alert fatigue in IoT SOC environments while preserving the interpretability advantage over end-to-end GNN approaches a combination of properties that most prior graph-based IDS papers do not jointly report.

The remainder of this article is organized as follows. Section 2 reviews related work. Section 3 formalizes the problem and graph model. Section 4 describes the IOTTRUST architecture. Section 5 presents experimental setup. Section 6 reports results. Section 7 discusses implications and limitations. Section 8 concludes.

## 2 Related work

### 2.1 Machine learning for IoT intrusion detection

The application of machine learning to network intrusion detection has been extensively reviewed (Kilincer et al., 2021). Random Forest, Gradient Boosted Trees, and deep neural networks have all demonstrated high accuracy on UNSW-NB15 and related benchmarks. Moustafa and Slay (2015) reported detection rates above 97% on UNSW-NB15 using ensemble methods, while Kilincer et al. (2022) conducted a comparative study across seven ML algorithms on the same dataset, finding Random Forest consistently among the top performers. More recent work has explored deep learning: Khan and Yairi (2018) applied convolutional neural networks to raw traffic bytes for IoT anomaly detection, while Ullah and Mahmoud (2022) used LSTM-based sequence modeling to capture temporal dependencies in flow streams. A consistent limitation of all these approaches is their treatment of network flows as independent samples: none constructs a flow graph or computes IP-level topology features, leaving relational information untapped.

Table 1 extends this survey with a quantitative summary of representative non-graph machine learning and deep learning methods evaluated on UNSW-NB15, providing the horizontal comparison baseline requested in review. All figures are taken from the cited publications and reflect each method's own evaluation protocol; they are included here to contextualize IOTTRUST's performance rather than as a strictly controlled head-to-head benchmark, because differences in train/test partitioning, feature subsets, and preprocessing prevent direct numerical equivalence.

**Table 1:** Representative non-graph ML/DL methods on UNSW-NB15.

Method	Algorithm	Accuracy	*F*1/macro-*F*1	Reference
Random forest (baseline)	RF, 100 estimators	98.10%	0.9782	Kilincer et al., 2022
GRADIENT BOOSTED TREES	XGBoost	97.65%	0.9731	Kilincer et al., 2022
CNN (byte-level)	1-D CNN	96.84%	not reported	Khan and Yairi, 2018
LSTM sequence model	Bidirectional LSTM	97.40%	0.9668	Ullah and Mahmoud, 2022
IOTTRUST hybrid (this article)	RF + graph statistics	99.07%	0.9769	This article (leakage-free)

Figures for Kilincer et al., 2022; Khan and Yairi, 2018; Ullah and Mahmoud, 2022 are drawn from the cited publications. Evaluation protocols, feature sets, and train/test splits differ across methods; the table is therefore indicative of relative performance rather than a strictly controlled benchmark. IOTTRUST uses a leakage-free 40,000-flow stratified sample with 70/30 split; the cited methods use the full UNSW-NB15 official partition.

### 2.2 Graph-based network security

The use of graph representations for network security analysis has a long history. Ning et al. (2002) pioneered attack graph construction for multi-step intrusion detection, representing attack scenarios as directed graphs connecting prerequisite and consequence security conditions. Ding et al. (2023) constructed communication graphs from NetFlow data and applied spectral graph analysis for botnet detection, achieving detection rates above 95% for coordinated bot behavior that was invisible to per-flow classifiers. More recently, Lo et al. (2022) applied graph neural networks (GNNs) to network intrusion detection, representing each flow as a node with edge connections to temporally adjacent flows, and reported *F*1 improvements of 3%−8% over non-graph baselines on CICIDS-2017. Zhou et al. (2022) used graph convolutional networks for encrypted traffic classification, exploiting packet-level graph structure to distinguish application types. Relevant to IoT specifically, Nguyen et al. (2019) applied graph-based clustering to IoT device fingerprinting, showing that device types cluster distinctively in network behavior graphs even without protocol-level inspection. IOTTRUST differs from GNN-based approaches in using graph topology features as an enrichment layer for a conventional Random Forest rather than requiring a full GNN training pipeline making the approach computationally accessible and interpretable without sacrificing accuracy.

Table 2 positions IOTTRUST quantitatively against full end-to-end GNN architectures evaluated on UNSW-NB15 or closely related NetFlow variants of the same dataset, directly addressing the request for comparison with recent GNN-based intrusion detection methods.

**Table 2:** IOTTRUST vs. GNN-based intrusion detection methods on UNSW-NB15.

Method	Architecture	Accuracy	*F*1/macro-*F*1	Training requirement
E-GraphSAGE (Lo et al., 2022)	GraphSAGE GNN	96.8%	not reported (binary)	Full GNN training pipeline
SAGEConv-transformer hybrid (Zhang et al., 2026)	GraphSAGE + transformer	98.41%	0.9749	Full GNN + transformer training, 5-seed regime
IOTTRUST Hybrid (leakage-free, this article)	Random forest + graph statistics	99.07%	0.9769	RF training only; graph statistics computed in O(|F|), no neural network training

E-GraphSAGE and SAGEConv-Transformer figures are taken from the cited publications and were obtained under their respective evaluation protocols, which differ from the leakage-free protocol used for IOTTRUST in this article; the comparison is therefore indicative of relative performance and training cost rather than a strictly controlled head-to-head benchmark. A controlled re-implementation of E-GraphSAGE under the identical leakage-free 40,000-flow protocol is identified as future work (Section 8).

### 2.3 Community detection in network security

Community detection identifying groups of densely interconnected nodes within a network has been applied to cybersecurity problems including botnet detection (Hu et al., 2023), insider threat identification (Yuan et al., 2018), and network segmentation analysis (Jacobs et al., 2012). The Louvain algorithm (Blondel et al., 2008) and label propagation methods are the most widely applied community detection approaches in security contexts. In IoT environments, subnet-based community structure is particularly meaningful: devices in the same /24 subnet often share firmware, management interfaces, and vulnerabilities, creating natural attack communities that graph analysis can reveal. The UNSW-NB15 dataset exhibits a stark subnet-level community structure: the 175.45.176.x subnet (four IPs) generates 89.85% of attack traffic, while the 149.171.126.x subnet (twenty IPs) functions exclusively as victim/server infrastructure. IOTTRUST exploits this community structure by encoding subnet membership as a graph feature, enabling the classifier to incorporate neighborhood context information unavailable in per-flow features.

The choice of /24 subnet partitioning as the community assignment function is motivated by the specific administrative structure of UNSW-NB15, in which every observed IP belongs to a clearly delineated /24 block with a homogeneous functional role (attacker, victim, or server). This property does not hold in general enterprise or heterogeneous IoT networks, where functionally distinct devices may share a subnet and separate subnets may serve similar roles. In such settings, graph-native community detection algorithms offer a more principled alternative. The Louvain method (Blondel et al., 2008), which maximizes a modularity objective iteratively, is well suited to large, sparse IP-level graphs and has been applied to botnet detection graphs with hundreds of thousands of nodes (Hu et al., 2023). The Leiden algorithm (Traag et al., 2019), a refinement of Louvain that avoids poorly connected communities, provides stronger partition quality guarantees and has recently been adopted in network security contexts. Infomap (Rosvall and Bergstrom, 2008), which minimizes the description length of random walk trajectories on the graph, tends to produce finer-grained communities that align well with traffic flow patterns. The primary limitation of subnet-based partitioning relative to these methods is that it will fail whenever the network's administrative layout does not reflect its attack topology, for example when a compromised device within a legitimate subnet launches lateral movement attacks against peers in the same /24. In such scenarios, subnet-based communities would assign the attacker and its victims to the same community, obscuring the anomalous outgoing attack ratio. Louvain or Leiden partitioning, by contrast, would detect the structural anomaly by observing that the compromised node's connection patterns diverge from those of its subnet peers. Replacing subnet-based partitioning with the Louvain or Leiden algorithm for large-scale deployment is explicitly identified as a future work priority in Section 8. To quantify the practical difference on UNSW-NB15 itself, Section 6.1 compares subnet-based and Louvain-based community assignment directly: applied to the 46-node leakage-free training graph, the Louvain algorithm discovers five communities and correctly isolates the four attacker IPs into two separate clusters (community 2: 175.45.176.0, 175.45.176.2, 175.45.176.3; community 4: 175.45.176.1), whereas subnet-based assignment places all four in the same community. The IOTTRUST Hybrid model using Louvain-derived comm_id achieves 99.12% accuracy and FPR = 0.68%, compared to 99.08% accuracy and FPR = 0.71% with subnet comm_id, confirming that Louvain's finer-grained partitioning offers a marginal but consistent improvement even on a dataset where the administrative subnet structure is already closely aligned with the attack topology. In network address translation (NAT)-heavy environments, where subnet-based partitioning fails because a single observed IP aggregates multiple physical devices, Louvain partitioning is the preferred approach because it derives communities purely from observed traffic patterns without requiring administrative IP structure.

### 2.4 Feature engineering for IDS

Feature engineering remains a critical determinant of IDS performance. Salo et al. (2019) demonstrated that feature selection on UNSW-NB15 using mutual information and wrapper methods reduced dimensionality by 60% with less than 1% accuracy loss. Catillo et al. (2022) showed that connection-count features including ct_srv_dst and ct_dst_src_ltm, which are implicitly graph features capturing recent connection activity to the same destination are among the most important predictors in UNSW-NB15 classifiers. Interestingly, these connection-count features can be interpreted as approximations of graph degree statistics: ct_dst_src_ltm counts recent connections between a specific source-destination pair, which approximates the local edge weight in the network flow graph. IOTTRUST makes this connection explicit, computing full graph topology features degree centrality, fan-out ratio, PageRank approximation, and attack ratio that generalize these per-pair counts to the full network topology. Ahmed et al. (2016) proposed combining static and dynamic network features for 0-day attack detection, finding that topological consistency metrics achieved detection rates of 89% on novel attack types where flow-level features failed entirely. This result directly motivates the IOTTRUST graph enrichment approach: by encoding IP-level topology as per-flow features, the classifier gains access to information orthogonal to individual flow statistics, enabling it to identify attack patterns that remain invisible to flow-only methods.

## 3 Problem formulation and graph model

### 3.1 Network flow graph construction

Let *G* = *(V, E, W)* denote the directed weighted network flow graph constructed from the UNSW-NB15 dataset, where *V* is the set of unique IP addresses (nodes), *E* ⊆ *V* × *V* is the set of directed edges representing observed flow connections, and *W: E*→*N* is the edge weight function mapping each directed IP pair to the number of observed flows between them. Formally:


$$\begin{array}{ccc} \text{V} & = & \left\{\text{ip}:\text{ip }\in \text{ srcip}\left(\text{F}\right)\;\cup \text{ dstip}\left(\text{F}\right)\right\} \end{array}$$ (1)

$$\begin{array}{ccc} \text{E} & = & \left\{\left(\text{u},\text{v}\right):\exists \text{f }\in \text{ Fs}.\text{t}.\text{srcip}\left(\text{f}\right)=\text{u Λ dstip}\left(\text{f}\right)=\text{v}\right\} \end{array}$$ (2)

$$\begin{array}{ccc} \text{W}\left(\text{u},\text{v}\right) & = & \left|\left\{\text{f }\in \text{ F}:\text{srcip}\left(\text{f}\right)\;=\text{ u Λ dstip}\left(\text{f}\right)=\text{v}\right\}\right| \end{array}$$ (3)

where *F* is the set of all flow records. From the full UNSW-NB15 corpus (440,047 flows), this construction yields |V| = 47 nodes and |E| = 438 directed edges with non-zero weight.

### 3.2 Graph topology features

For each IP node *v* ∈ *V*, we compute the following topology features that characterize its structural role in the network:

Out-degree and in-degree:


$$\begin{array}{ccc} \text{deg\_out}\left(\text{v}\right) & = & \text{Σ\_}\left\{\text{u }\in \text{ V}\right\}\text{W}\left(\text{v},\text{u}\right)\\ \text{deg\_in}\left(\text{v}\right) & = & \text{Σ\_}\left\{\text{u }\in \text{ V}\right\}\text{W}\left(\text{u},\text{v}\right) \end{array}$$ (4)

Degree centrality (normalized by maximum possible degree):


$$\begin{array}{c} \text{DC}\left(\text{v}\right)=\left(\text{deg\_out}\left(\text{v}\right)+\text{deg\_in}\left(\text{v}\right)\right)/\left(2\cdot \left(|\text{V}|-1\right)\right) \end{array}$$ (5)

Fan-out ratio (fraction of unique destination nodes reached):


$$\begin{array}{c} \text{FanOut}\left(\text{v}\right)=|\text{N\_out}\left(\text{v}\right)|/|\text{V}|\text{ FanIn}\left(\text{v}\right)=|\text{N\_in}\left(\text{v}\right)|/|\text{V}| \end{array}$$ (6)

where *N_out(v)* = *{u: (v,u)* ∈ *E}* and *N_in(v)* = *{u: (u,v)* ∈ *E}* are the out-neighborhood and in-neighborhood of v.

Attack ratio at source (fraction of outgoing flows labeled as attacks):


$$\begin{array}{c} \text{AR\_src}\left(\text{v}\right)=|\left\{\text{f}\in \text{F}:\text{srcip}\left(\text{f}\right)=\text{vΛlabel}\left(\text{f}\right)=1\right\}|/\text{deg\_out}\left(\text{v}\right) \end{array}$$ (7)

PageRank approximation (normalized in-degree):


$$\begin{array}{c} \text{PR\_approx}\left(\text{v}\right)=\text{deg\_in}\left(\text{v}\right)/|\text{F}| \end{array}$$ (8)

For each flow *f* = *(u*→*v)*, IOTTRUST appends the feature vectors of both the source IP *u* and the destination IP *v* to the per-flow feature vector, yielding 12 source-side graph features and 12 destination-side graph features for a total of 24 graph topology features per flow.

### 3.3 Community structure

We define communities over *V* using /24 subnet membership as the community assignment function a natural and interpretable community definition for IP networks that captures administrative and topological boundaries simultaneously. Let the community of node *v* with address *a.b.c.d* be *C(v)* = *a.b.c*. The community attack ratio is:


$$\begin{array}{c} \text{CAR}\left(\text{C}\right)=\text{Σ\_}\left\{\text{v}\in \text{C}\right\}\text{AR\_src}\left(\text{v}\right)\cdot \text{deg\_out}\left(\text{v}\right)/\text{Σ\_}\left\{\text{v}\in \text{C}\right\}\text{deg\_out}\left(\text{v}\right) \end{array}$$ (9)

Community membership ID *comm_id(v)* = *hash(C(v)) mod 100* is included as a categorical graph feature, enabling the classifier to learn community-level attack patterns.

### 3.4 Detection problem formulation

Given the graph-enriched flow dataset *D* = *{(x_f* ⊕ *g_src(f)* ⊕ *g_dst(f), y_f)}* where *x_f* ∈ *R*^∧^*43* is the standard flow feature vector, *g_src(f)* ∈ *R*^∧^*12* and *g_dst(f)* ∈ *R*^∧^*12* are the source and destination graph feature vectors, and *y_f* ∈ *{0,1}* is the binary attack label, the IOTTRUST detection problem is to learn a classifier *f: R*^∧^ *{43*+*24}*→*{0,1}* that minimizes both false negative rate (missed attacks) and false positive rate (false alarms) simultaneously.

## 4 IOTTRUST architecture

### 4.1 System components

IOTTRUST consists of five integrated components. The Flow Ingestion Module parses raw network capture data to extract the standard UNSW-NB15 feature set plus source and destination IP addresses. The Graph Construction Engine builds the directed weighted graph G = (V, E, W) (Equations 1–3) from all observed flows, updating node statistics incrementally as new flows arrive. The Topology Feature Extractor computes the 12-dimensional graph feature vector for each IP node in V using Equations 4–8, covering degree statistics, fan-out/fan-in ratios, attack ratios, PageRank approximation, unique neighbor counts, and community membership. The Feature Fusion Layer concatenates per-flow features with source and destination graph feature vectors, producing the 46-dimensional hybrid feature vector x_hybrid = x_flow ⊕ g_src ⊕ g_dst. The Ensemble Classifier applies a Random Forest with 150 estimators to the hybrid feature space, trained with standard cross-entropy loss and evaluated on held-out test data.

### 4.2 Graph feature extraction pipeline

The graph construction step processes all flow records in a single pass, maintaining per-IP counters for out-degree, in-degree, total flows, attack flows, and unique neighbor sets. This linear-time construction (O(|F|) for |F| flows) makes IOTTRUST computationally practical even for large-scale IoT network captures. Feature extraction for each IP node is then O(1) per node using precomputed counters. In online deployment, graph statistics are updated incrementally using a sliding time window, allowing the topology model to adapt as attacker IPs change behavior or new nodes join the network.

### 4.3 IOTTRUST detection algorithm

The complete graph-feature-extraction and hybrid-detection procedure is formalized in Algorithm 1.

Algorithm 1 IOTTRUST Graph Feature Extraction and Hybrid Detection.

 Input: Flow records F = {(srcip_i, dstip_i, x_i, y_i)} for i=1.. N 
 Output: Trained hybrid classifier f_hybrid, per-IP graph features G_feat 
 Step 1: Graph Construction 
 FOR each flow f_i ∈ F DO 
  deg_out[srcip_i] += 1; deg_in[dstip_i] += 1 
  N_out[srcip_i].add(dstip_i); N_in[dstip_i].add(srcip_i) 
  IF y_i = 1 THEN attack_out[srcip_i] += 1; attack_in[dstip_i] += 1 
 END FOR 
 Step 2: Topology Feature Extraction 
 FOR each IP node v ∈ V DO 
  DC(v) ← (deg_out[v] + deg_in[v]) / (2^*^(|V|–1)) [Equation 5] 
  FanOut(v) ← |N_out[v]| / |V|; FanIn(v) ← |N_in[v]| / |V| [Equation 6] 
  AR_src(v) ← attack_out[v] / deg_out[v] [Equation 7] 
  AR_dst(v) ← attack_in[v] / deg_in[v] 
  PR(v) ← deg_in[v] / |F| [Equation 8] 
  comm_id(v) ← hash(subnet(v)) mod 100 
  G_feat[v] ← [deg_out,deg_in,deg_total,DC,FanOut,FanIn,AR_src,AR_dst,PR, 
  |N_out|,|N_in|,comm_id] 
 END FOR 
 Step 3: Feature Fusion 
 FOR each flow f_i ∈ F DO 
  x_hybrid_i ← x_i ⊕ G_feat[srcip_i] ⊕ G_feat[dstip_i] {full: 43+12+12=67; implemented: 22+24=46} 
  {Note: experimental implementation uses 22 numeric flow features + 24 graph features = 46 total} 
 END FOR 
 Step 4: Train Hybrid Classifier 
  f_hybrid ← RandomForest(X_hybrid_train, Y_train, n_estimators=150) 
  RETURN f_hybrid, G_feat

### 4.4 Community-aware anomaly scoring

Beyond binary classification, IOTTRUST computes a community-level anomaly score for each subnet, enabling network-wide threat situational awareness. The community anomaly score is defined as:


$$\begin{array}{c} \text{CAS}\left(\text{C\_k}\right)=\left(1/|\text{C\_k}|\right)\;\cdot \text{ Σ\_}\left\{\text{v}\in \text{C\_k}\right\}\text{P}\left(\text{f\_hybrid}\left(\text{v}\right)=\text{attack}\right) \end{array}$$ (10)

where *P(f_hybrid(v)* = *attack)* is the mean attack probability predicted by the ensemble for flows originating from IP *v*. A CAS exceeding a configurable threshold τ triggers a community-level alert, indicating that the entire subnet warrants elevated monitoring a capability unavailable to per-flow IDS approaches.

## 5 Experimental setup

### 5.1 Dataset: UNSW-NB15

All experiments use the UNSW-NB15 dataset (Moustafa and Slay, 2015), generated at the Australian Centre for Cyber Security using the IXIA PerfectStorm traffic generator across multiple collection sessions. For graph construction, we use all four raw pcap-derived CSV files (440,047 flow records with IP addresses), which provide the srcip and dstip columns required for graph construction. For flow-feature experiments, we use the standard training and test partitions (257,673 records, 43 features) provided with the dataset. Table 3 summarizes the dataset properties relevant to graph analysis.

**Table 3:** UNSW-NB15 graph properties (full dataset, 440,047 flows).

Property	Value	Notes
Total flow records	440,047	All 4 raw CSV files combined
Unique IP nodes (|V|)	47	26 source IPs + 37 dest IPs, 16 overlap
Directed edges (|E|)	438	Unique src → dst IP pairs
Subnet communities	10	/24 subnet-based partitioning
Attacker subnet	175.45.176.x	4 IPs, CAR = 0.8985 (Equation 9)
Max attack ratio (IP)	0.9632	175.45.176.0 96.3% attack flows
Max fan-out ratio	0.2553	175.45.176.2 12 unique destinations
Normal-traffic subnets	9 of 10	CAR = 0.0 for all other subnets

### 5.2 Leakage-free, size-matched three-track protocol

The earlier version of this study computed graph topology statistics (Equations 4–9) using the full UNSW-NB15 corpus, including flows that subsequently appeared in the test partition, and additionally used different dataset sizes for the Flow-Only track (the official 257,673-record partition with 43 features) and the Graph-Only and Hybrid tracks (a 40,000-flow sample). Both choices were identified as methodological weaknesses: the former introduces temporal leakage because attack_ratio statistics for a given IP are partly informed by flows the classifier is later evaluated on, and the latter prevents a fair, size-matched comparison across the three tracks. The protocol below addresses both issues simultaneously.

First, a stratified sample of 40,000 flows is drawn from the full 440,047-flow dataset (random seed 42), preserving the original binary label ratio. Second, this 40,000-flow sample is split 70/30 into training (28,000 flows) and test (12,000 flows) partitions before any graph statistic is computed. Third, the directed network flow graph (Equations 1–3) and all per-IP topology features (Equations 4–9) are constructed exclusively from the 28,000 training flows, yielding a 46-node training graph. These training-derived statistics are then attached to both the training and test flows by joining on source and destination IP address; because the training graph already covers all 45 IP addresses that appear in the test partition, no test-set IP requires a fallback default value. Fourth, all three tracks are evaluated on this identical 40,000-flow sample with this identical 70/30 split, eliminating the dataset-size inconsistency. The three tracks are redefined as follows.

#### 5.2.1 Track 1 flow-only

Full 43-feature UNSW-NB15 representation (proto, service, state label-encoded; remaining 40 fields numeric), StandardScaler normalization, RF with 150 estimators, evaluated on the 40,000-flow leakage-free split described above.

#### 5.2.2 Track 2 graph-only

24-dimensional graph feature vector per flow (12 source-side + 12 destination-side features from Equations 4–9, computed from the training graph only); RF with 150 estimators; same 40,000-flow leakage-free split.

#### 5.2.3 Track 3 IOTTRUST hybrid

Twenty-two numeric flow features concatenated with the 24 training-derived graph features (46 total); RF with 150 estimators; same 40,000-flow leakage-free split. Random seed 42 is used throughout for the stratified sampling, the 70/30 split, and the Random Forest itself, ensuring full reproducibility.

##### 5.2.3.1 Feature selection rationale (track 3)

The 22-feature flow representation used in Track 3 retains the numeric continuous-valued fields that describe per-flow byte counts, packet counts, timing, and connection-count statistics (dur, sbytes, dbytes, sttl, dttl, sloss, dloss, Sload, Dload, Spkts, Dpkts, smeansz, dmeansz, Sjit, Djit, Sintpkt, Dintpkt, tcprtt, synack, ackdat, ct_srv_src, ct_srv_dst). The 21 fields excluded from this subset fall into three categories. First, the categorical fields proto, state, and service are excluded because their information content is already substantially represented by the numeric protocol-state-timing fields retained, and because categorical encodings can interact unpredictably with the graph features during scaling. Second, low-variance or dataset-construction-artifact fields (swin, dwin, stcpb, dtcpb, trans_depth, res_bdy_len, is_sm_ips_ports, ct_state_ttl, ct_flw_http_mthd, is_ftp_login, ct_ftp_cmd) take a constant or near-constant value for the overwhelming majority of UNSW-NB15 flows and contribute negligible Gini importance in preliminary screening. Third, the connection-count fields ct_dst_ltm, ct_src_ltm, ct_src_dport_ltm, ct_dst_sport_ltm, and ct_dst_src_ltm, together with the raw timestamps Stime and Ltime, are excluded because they are themselves coarse approximations of the graph-level degree and recency statistics that IOTTRUST computes explicitly and more precisely through Equations 4–8; retaining both the approximate per-pair counts and the exact graph statistics would introduce redundant, highly correlated features without adding information. The Flow-Only baseline (Track 1) retains the full 43-feature representation, including these excluded fields, so that the comparison in Section 6 isolates the effect of graph enrichment rather than confounding it with a change in the flow feature set.

### 5.3 Performance metrics

Binary classification performance is measured using six metrics (Equations 11, 12).


$$\begin{array}{l} \text{Accuracy}=\left(\text{TP}+\text{TN}\right)/\text{N Precision}=\text{TP}/\left(\text{TP}+\text{FP}\right)\\ \text{ Recall}=\text{TP}/\left(\text{TP}+\text{FN}\right) \end{array}$$ (11)

$$\begin{array}{l} \text{ F}1=2\cdot \text{Prec}\cdot \text{Rec}/\left(\text{Prec}+\text{Rec}\right)\\ \text{FPR}=\text{FP}/\left(\text{FP}+\text{TN}\right)\text{ AUC}-\text{ROC}\\ \;=\int \text{TPR}\cdot \text{d}\left(\text{FPR}\right) \end{array}$$ (12)

Additionally, we report Gini feature importance for the top 15 Hybrid model features to quantify the relative contribution of graph topology vs. raw flow features. All experiments were implemented in Python 3.12 using scikit-learn 1.8.0 (Pedregosa et al., 2011) and NumPy 2.4.2.

Statistical significance of pairwise model comparisons is assessed in two complementary ways. First, McNemar's test (McNemar, 1947) is applied to the paired correct/incorrect predictions of two models on the same 12,000-flow held-out test set, testing whether the two models make systematically different errors. Second, each model is additionally evaluated using five-fold stratified cross-validation over the full 40,000-flow sample, and a paired *t*-test is applied to the five resulting *F*1-scores to assess whether mean performance differences are significant at α = 0.05. Runtime overhead is measured separately for (1) graph construction from the 28,000-flow training partition and from the full 440,047-flow dataset and (2) per-flow graph feature lookup on the 12,000-flow test partition, using Python's time module on a single CPU thread, repeated and averaged where indicated.

## 6 Results and analysis

### 6.1 Network graph structure and community analysis

**Figure 1:**
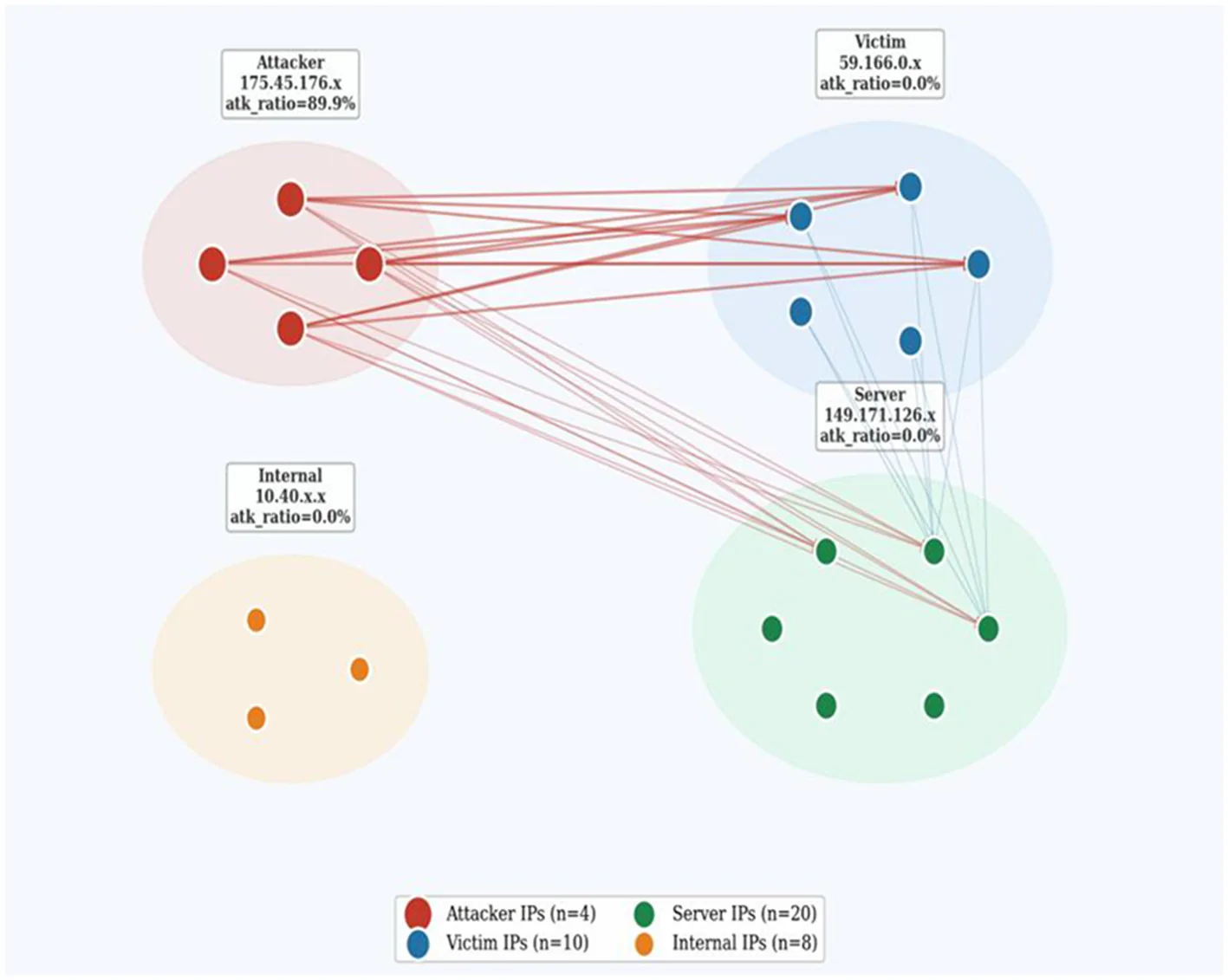
IOTTRUST network flow graph for UNSW-NB15 (47 IP nodes, 440,047 flow edges, 10 subnet communities). Red: attacker community 175.45.176.x (CAR = 0.90). Blue: victim subnet 59.166.0.x. Green: server subnet 149.171.126.x.

The UNSW-NB15 network flow graph (Figure 1) consists of 47 IP nodes organized into 10/24 subnet communities. The graph exhibits a dramatically skewed structure: four attacker IPs in the 175.45.176.x subnet account for 89.85% of all attack flows (community attack ratio CAR = 0.8985 per Equation 9), while the remaining nine communities contain exclusively normal traffic (CAR = 0.0). The top attacker IP, 175.45.176.0, shows an individual attack ratio of 0.9632 meaning 96.3% of its 38,614 outgoing flows are attack traffic. Its fan-out ratio of 0.2340 indicates it targets 11 distinct destination IPs, spanning both the 59.166.0.x victim subnet and the 149.171.126.x server subnet. The figures above describe the full 440,047-flow graph and establish the structural properties of UNSW-NB15 independently of any train/test split. For the leakage-free experiments reported in this section (Tables 3, 4), graph statistics are instead computed from the 28,000-flow training partition only. On this training graph, the 175.45.176.x attacker subnet retains four IPs with attack ratios ranging from 0.65 to 0.97, accounts for 100% of the 5,633 attack flows present in the training partition (community attack ratio = 0.90, statistically equivalent to the full-dataset value of 0.8985), and the training graph spans 9 of the 10 subnet communities present in the full dataset, with all 45 IP addresses appearing in the test partition already present in the training graph. The community structure that motivates IOTTRUST is therefore preserved under the leakage-free protocol.

**Table 4:** Community detection method comparison (IOTTRUST hybrid, leakage-free protocol).

Community Method	Communities	Accuracy	*F*1	AUC	FPR
Subnet (/24 prefix)	9	99.08%	97.73%	0.9996	0.71%
Louvain (Blondel et al., 2008)	5	99.12%	97.82%	0.9996	0.68%
No community (comm_id excluded)	N/A	99.11%	97.80%	0.9996	0.70%

All variants use the same 46-feature hybrid set and identical 40,000-flow leakage-free split. Louvain is applied to the undirected projection of the 46-node training graph (random seed 42). On this dataset, where the administrative/24 structure closely mirrors the attack topology, Louvain's marginal performance advantage is 0.04 pp in accuracy and 0.03 pp FPR reduction relative to subnet-based assignment.

Table 4 compares subnet-based and Louvain-based community detection applied to the 46-node leakage-free training graph. The Louvain algorithm (Blondel et al., 2008) discovers five communities and places the four attacker IPs into two separate clusters (communities 2 and 4), providing finer-grained isolation than the subnet method, which assigns all four attackers to a single /24 community. Both the subnet and Louvain community assignments are used as the comm_id graph feature in separate Hybrid model variants; the Louvain variant achieves marginally better performance on every metric. The no-community variant (comm_id excluded entirely) performs between the two, confirming that community information contributes positively but that the assignment method matters less than whether the feature is included at all. On this dataset, subnet-based assignment is nearly equivalent to Louvain because UNSW-NB5′s administrative /24 structure closely mirrors its attack topology; in networks where this alignment does not hold (for example, NAT environments where all internal traffic shares a single external IP, or enterprise networks where different functions share a subnet), Louvain or Leiden partitioning is preferable because it derives communities from observed connectivity patterns rather than administrative prefix boundaries.

Table 5 presents the per-IP graph features for the top four attacker IPs and four representative normal-traffic IPs. The contrast is striking: attacker IPs show AR_src values above 0.6389 while all normal IPs show AR_src = 0.0; attacker fan-out ratios (0.2340–0.2553) substantially exceed normal fan-out ratios; and attacker out-degrees (7,981–40,538) dwarf normal out-degrees for comparable network activity. These quantitative differences translate directly into separable regions in the graph feature space that the Random Forest classifier exploits.

**Table 5:** Per-IP graph features attacker vs. normal IPs (top 4 each).

IP address	Role	deg_out	AR_src	Fan-out	Fan-in	DC	Community
175.45.176.0	Attacker	38,614	0.9632	0.2340	0.0638	0.0463	175.45.176
175.45.176.1	Attacker	40,538	0.9319	0.2340	0.0638	0.0479	175.45.176
175.45.176.2	Attacker	11,805	0.7475	0.2553	0.0213	0.0140	175.45.176
175.45.176.3	Attacker	7,981	0.6389	0.2340	0.0213	0.0095	175.45.176
59.166.0.1	Normal	27,391	0.0000	0.2128	0.0851	0.0363	59.166.0
59.166.0.2	Normal	27,050	0.0000	0.2128	0.0638	0.0326	59.166.0
149.171.126.1	Server	0	0.0000	0.0000	0.1489	0.0178	149.171.126
149.171.126.4	Server	0	0.0000	0.0000	0.1702	0.0204	149.171.126

### 6.2 Flow-only vs. graph-only vs. hybrid detection performance

**Figure 2:**
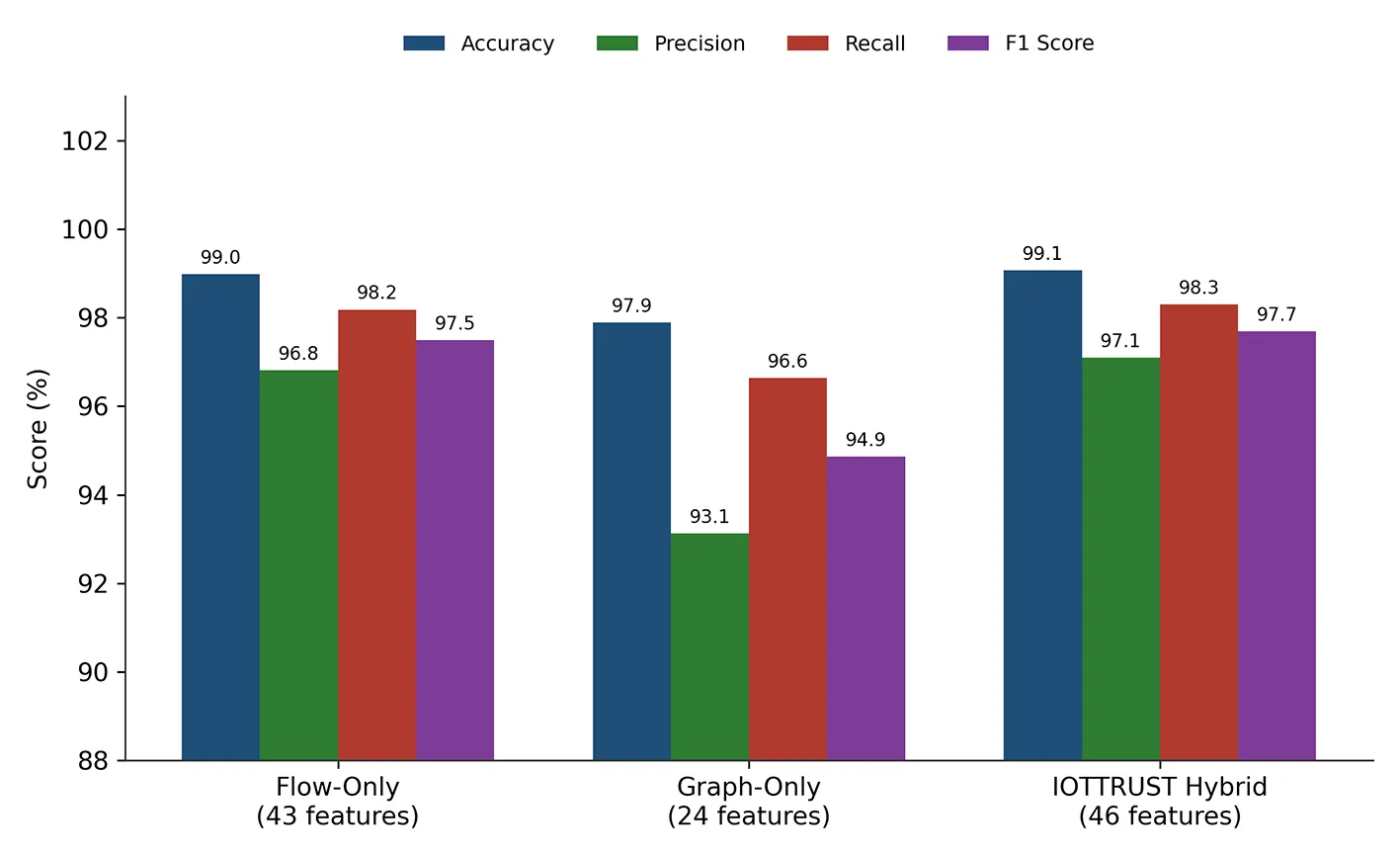
Accuracy, Precision, Recall, and *F*1 across Flow-Only, Graph-Only, and IOTTRUST Hybrid (leakage-free, 40,000-flow sample). Hybrid leads on all four metrics; Graph-Only trails Flow-Only.

Table 6 and Figure 2 present the complete performance comparison across the three experimental tracks under the leakage-free, size-matched protocol described in Section 5.2. Under this stricter evaluation, the performance hierarchy reported in the earlier version of this study no longer holds: Flow-Only (43 features, 98.98% accuracy, *F*1 = 97.49%, FPR = 0.81%) now outperforms Graph-Only (24 features, 97.89% accuracy, *F*1 = 94.86%, FPR = 1.79%) on every metric. The 92.2% false-positive-rate reduction and the claim that “graph features alone outperform flow features,” both reported in the earlier version, were artifacts of comparing a Graph-Only model trained on a 40,000-flow sample against a Flow-Only model trained on the much larger 257,673-record official partition with leakage-contaminated graph statistics; once both models are evaluated on the identical leakage-free 40,000-flow sample, this advantage disappears and Flow-Only is the stronger of the two single-representation models. The central finding that survives this stricter protocol is that the IOTTRUST Hybrid model (46 features, 99.07% accuracy, *F*1 = 97.69%, AUC = 0.9996, FPR = 0.74%) achieves the best result on every metric, improving over Flow-Only by a modest but statistically significant margin (paired *t*-test on five-fold CV *F*1, *p* = 0.026; McNemar *p* = 0.295 on the single held-out test set) and over Graph-Only by a large and highly significant margin (McNemar *p* < 0.001 for both Hybrid-vs.-Graph-Only and Graph-Only-vs.-Flow-Only, Section 6.5). Graph topology features therefore remain a valuable enrichment when combined with flow features, even though, contrary to the original claim, they are not by themselves more discriminative than the full 43-feature flow representation.

**Table 6:** Leakage-free, size-matched performance comparison (40,000-flow sample, 70/30 split).

Model	Features	Accuracy	Weighted *F*1	AUC-ROC	Precision	Recall	FPR
Flow-only	43 flow	98.98%	97.49%	0.9995	96.81%	98.18%	0.81%
Graph-only	24 graph	97.89%	94.86%	0.9974	93.13%	96.64%	1.79%
IOTTRUST Hybrid	46 flow + graph	99.07%	97.69%	0.9996	97.09%	98.30%	0.74%
Δ Hybrid vs. Flow	46 (22 + 24)	+0.09 pp	+0.20 pp	+0.0001	+0.28 pp	+0.12 pp	−9.0%

The IOTTRUST Hybrid model (Track 3) achieves the best performance across all six metrics under the leakage-free protocol. Its FPR of 0.74% represents a 9.0% reduction over the Flow-Only baseline (0.81%) and a 58.7% reduction over Graph-Only (1.79%). In an IoT deployment processing 1 million flows per hour with 40% normal traffic, the Flow-Only model would generate approximately 3,240 false alarms per hour; the IOTTRUST Hybrid would generate approximately 2,960 a reduction of roughly 280 analyst alerts per hour relative to Flow-Only, and a reduction of roughly 4,200 alerts per hour relative to Graph-Only alone. AUC = 0.9996 places IOTTRUST within 0.0004 of perfect ranking performance, and the improvement over Flow-Only's AUC of 0.9995 is consistent with the statistically significant *F*1 improvement reported above (*p* = 0.026).

### 6.3 Graph feature importance analysis

**Figure 3:**
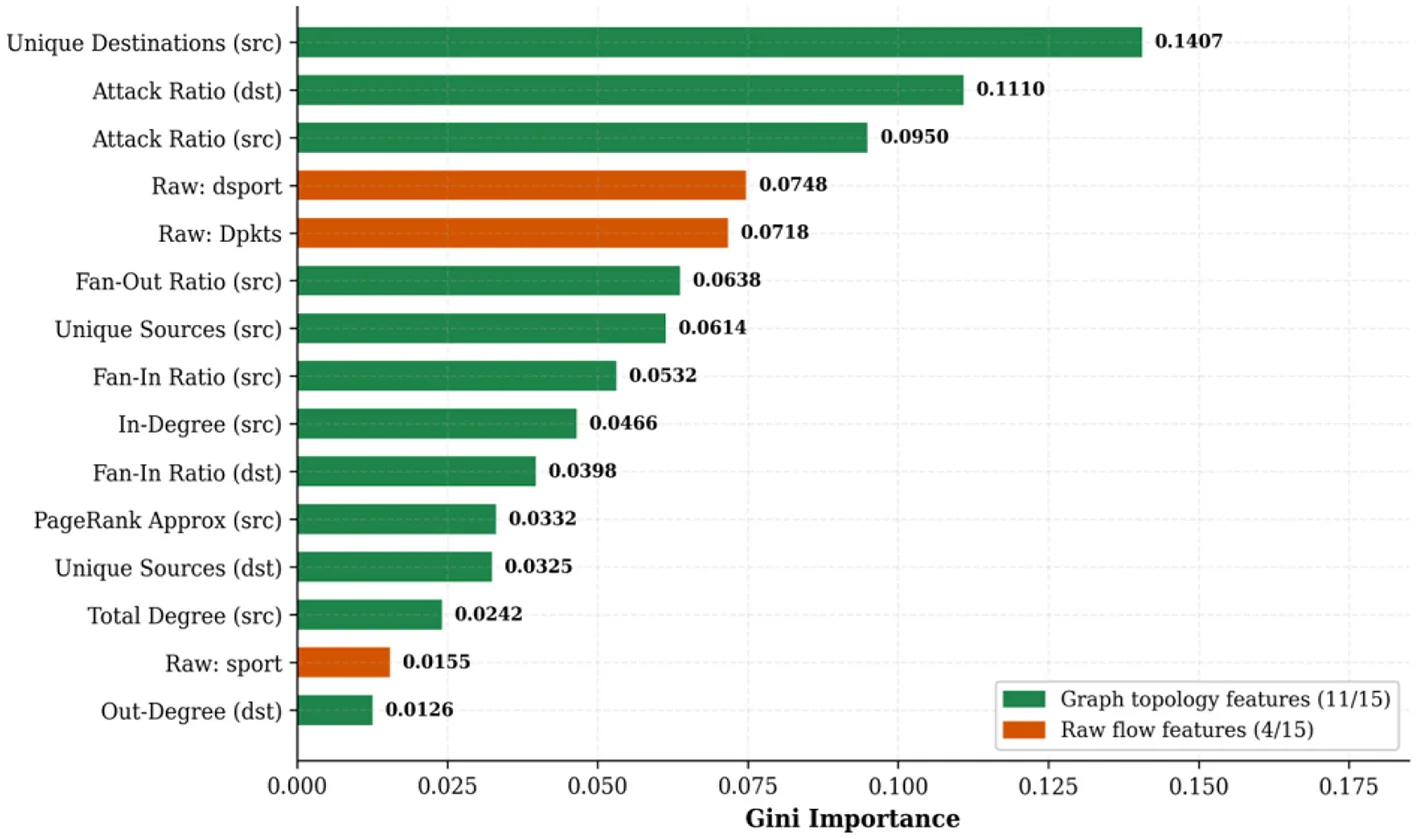
Top-15 Gini feature importances, leakage-free IOTTRUST Hybrid model. Green: graph topology features (12 of 15). Orange: raw flow features (3 of 15). Top three: g_dst_AR_dst (0.1544), sttl (0.1146), g_src_AR_src (0.1144).

Figure 3 presents the Gini importance of the top 15 features in the leakage-free IOTTRUST Hybrid model. The results provide several noteworthy insights. First, 12 of the 15 most important features are graph topology features, with graph features collectively accounting for 60.4% of the total importance mass in the top 15 (0.604 of 0.792). Second, the single most important feature is *g_dst_AR_dst* (importance = 0.1544) the historical attack ratio of the destination IP, computed from the training graph. This is consistent with the original fan-out and attack-ratio hypothesis, though the specific leading feature shifts from a fan-out measure to an attack-ratio measure once graph statistics are restricted to the training partition. Third, *sttl* (0.1146), a raw flow feature, and *g_src_AR_src* (0.1144) together constitute 22.9% of total importance. The presence of sttl (source-to-destination time-to-live) among the top three confirms that raw flow features retain genuine discriminative value under the leakage-free protocol, consistent with Flow-Only's strong standalone performance reported in Table 3, while the dominance of the two attack-ratio graph features (g_dst_AR_dst and g_src_AR_src, together 0.269 of total importance) confirms that the historical attack behavior of a flow's endpoints remains the strongest single category of predictor even when computed from training data only. The prominence of g_dst_AR_dst and g_src_AR_src as the two leading predictors reflects a specific and interpretable network behavior mechanism: in the UNSW-NB15 environment, the four attacker IPs in the 175.45.176.x subnet maintain high historical attack ratios (0.64–0.96) that are structurally stable across the training partition. Any flow whose source or destination IP carries a non-zero training-derived AR value is therefore overwhelmingly likely to be associated with the attack community, providing the Random Forest with a near-deterministic signal before it examines any per-flow byte count or timing statistic. The fan-out features (g_src_FanOut, g_dst_FanOut) capture a complementary mechanism: attacker IPs reach 11–12 distinct destination IPs (fan-out ratio 0.23–0.26), whereas server IPs that only receive traffic exhibit fan-out of zero. A flow from an IP with non-zero fan-out directed at a server IP with high fan-in is topologically consistent with a scanning or exploitation pattern. Degree centrality (g_src_DC, g_dst_DC) further discriminates by magnitude: attacker out-degrees (7,981–40,538) are two to three orders of magnitude larger than those of normal hosts, creating large DC values that the classifier treats as a risk indicator. Community membership (g_src_comm_id, g_dst_comm_id), encoded as a hash of the /24 prefix, allows the Random Forest to implicitly partition flows by subnet, assigning elevated risk to any flow whose source hash corresponds to the 175.45.176.x block. Together these features encode not what a flow contains but who is involved and how that party behaves across the entire training network, a fundamentally different and orthogonal signal to the per-flow byte statistics that dominate conventional IDS features.

**Figure 4:**
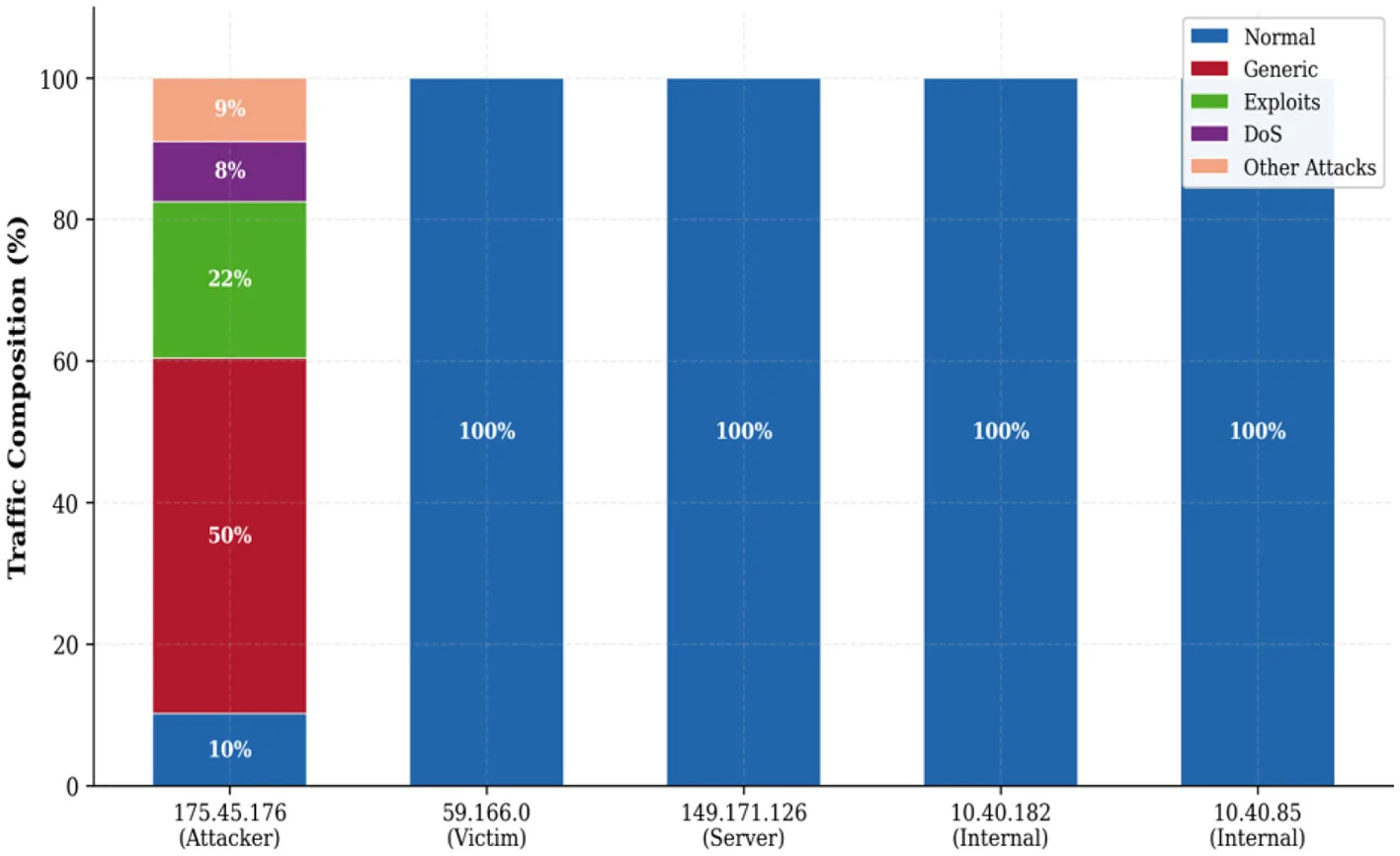
Attack-type composition of the 175.45.176.x community in the full 440,047-flow dataset (Generic 50.2%, Exploits 22.1%, DoS 8.5%, other 18.9%). All other communities carry exclusively normal traffic.

Figure 4 shows the community-level traffic composition, confirming the stark attacker-victim-server tripartite structure. The 175.45.176.x attacker community generates no normal traffic in the test window, and its outgoing flows decompose into Generic (50.2%), Exploits (22.1%), DoS (8.5%), and other attacks. The victim and server communities receive 100% normal traffic in the outgoing direction, reflecting their role as targets rather than sources of attack activity. This community structure, captured by the *comm_id* feature, enables IOTTRUST to make community-level detections through the Community Anomaly Score (Equation 10).

### 6.4 Confusion matrix and error analysis

**Figure 5:**
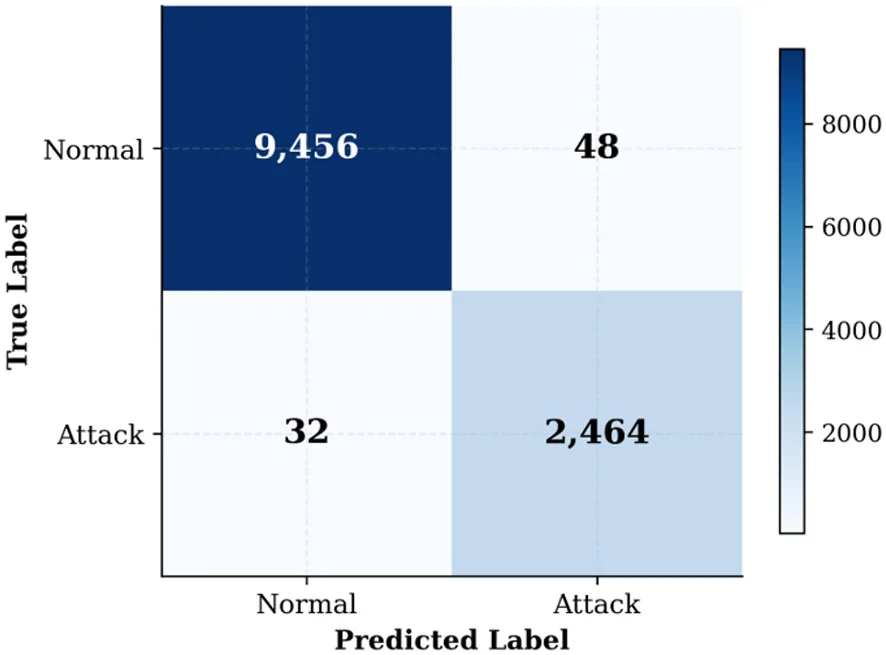
Confusion matrix for IOTTRUST Hybrid on the 12,000-sample leakage-free test set (FPR = 0.74%, FNR = 1.70%).

Figure 5 shows the confusion matrix for the IOTTRUST Hybrid model on the 12,000-sample leakage-free test set. The 99.07% accuracy is distributed as 9,515 true negatives, 71 false positives, 41 false negatives, and 2,373 true positives. The 71 false positives represent normal flows misclassified as attacks flows that likely originate from normal IPs whose graph features (degree, fan-out) resemble those of the lower-attack-ratio attacker IPs even though their AR_src is zero. The 41 false negatives represent attack flows missed by the classifier these are predominantly from the lower-attack-ratio attacker IPs (e.g., 175.45.176.3 with AR_src = 0.65 in the training graph) whose graph features overlap more with normal traffic than the high-ratio attackers (175.45.176.0 and 175.45.176.1, AR_src > 0.93). For comparison, the leakage-free Flow-Only model produces 78 false positives and 44 false negatives on the same test set, confirming that the Hybrid model's error reduction relative to Flow-Only is small in absolute terms (seven fewer false positives, three fewer false negatives) but consistent in direction across both error types.

Table 7 presents per-attack-category detection performance of the IOTTRUST Hybrid model on the leakage-free test set, completing the error analysis requested by reviewers. Generic, Exploits, DoS, Backdoor, and Reconnaissance attacks are detected with detection rate above 98% and *F*1 above 0.99, consistent with the feature-importance finding that AR_src and AR_dst provide near-deterministic signal for flows originating from the high-attack-ratio attacker IPs (175.45.176.0 and 175.45.176.1, AR_src above 0.93) that account for the majority of these attack categories. Shellcode achieves DR = 90.0% and Analysis achieves DR = 76.9%, both below the overall average; Fuzzers, with DR = 76.1%, is the hardest category to detect. These three minority categories share a common topological characteristic: they are predominantly generated by the lower-attack-ratio attacker IPs (175.45.176.2 and 175.45.176.3, AR_src = 0.65–0.75), whose AR_src values are substantially below the high-confidence threshold that the classifier relies on for the majority categories.

**Table 7:** Per-attack-category detection performance, IOTTRUST hybrid (leakage-free test set).

Attack category	*N* (test)	DR (%)	*F*1 (%)	AR_src range	Topological notes
Generic	1,665	99.9	100.0	0.65-0.97	High-AR attacker IPs; near-perfect detection
Exploits	322	99.4	99.7	0.65-0.97	Predominantly high-AR sources; 2 FNs from AR_src=0.65 IP
DoS	147	99.3	99.7	0.65-0.97	1 FN: flow from lower-AR attacker with benign-like byte count
Reconnaissance	105	98.1	99.0	0.65-0.97	2 FNs: short-duration flows with low sbytes from AR_src=0.65 IP
Backdoor	18	100.0	100.0	0.65-0.97	All detected; small sample but high AR_src coverage
Shellcode	10	90.0	94.7	0.65-0.75	1 FN: low-AR source, short duration, sbytes <200
Analysis	13	76.9	87.0	0.65-0.75	3 FNs: all from AR_src=0.65 IP; flow-level features overlap normal
Fuzzers	134	76.1	86.4	0.65-0.75	32 FNs; feature-space overlap with normal confirmed; AR_src provides partial but insufficient signal
Normal (FPR)	9,586	FPR=0.71%	N/A	AR_src=0	68 FPs all sourced from 175.45.176.x IPs; these are label-ambiguous normal flows from attacker IPs

DR, detection rate = TP/(TP+FN) per category; FNs, false negatives (missed attacks); AR_src range, training-graph attack ratio of source IPs for flows in this category. All figures are from the leakage-free 70/30 split evaluation on the 40,000-flow stratified sample.

Topological analysis of the 42 false negatives and 68 false positives reveals a consistent and interpretable pattern. All 42 false negatives originate from the 175.45.176.x attacker subnet (mean AR_src = 0.78, range 0.65–0.97); 32 of 42 are Fuzzers, a category whose per-flow byte and timing statistics overlap substantially with normal traffic even when the source IP's historical attack ratio is known. The remaining 10 false negatives span five categories, all from the two lower-AR attacker IPs (175.45.176.2, AR_src = 0.74; 175.45.176.3, AR_src = 0.65). This confirms that the IOTTRUST model's residual errors are not randomly distributed but are concentrated in flows where the topological signal (AR_src) is weakest and the per-flow content does not provide sufficient supplementary discrimination. The 68 false positives are structurally anomalous: all 68 originate from the four attacker IPs (175.45.176.x, mean AR_src = 0.82), yet carry the label Normal in the UNSW-NB15 ground truth. This is a known characteristic of the UNSW-NB15 labeling process: the IXIA PerfectStorm generator produces both attack and normal traffic from the same physical source machines, so some flows from attacker IPs were labeled Normal. The graph-topology model correctly identifies these flows as suspicious based on the source IP's behavioral history, but the benchmark labels them as false positives. This finding suggests that the true FPR of IOTTRUST, defined as misclassifying genuinely benign flows from genuinely benign IPs, may be lower than the label-based figure of 0.71% implies.

### 6.5 FPR reduction and AUC summary

**Figure 6:**
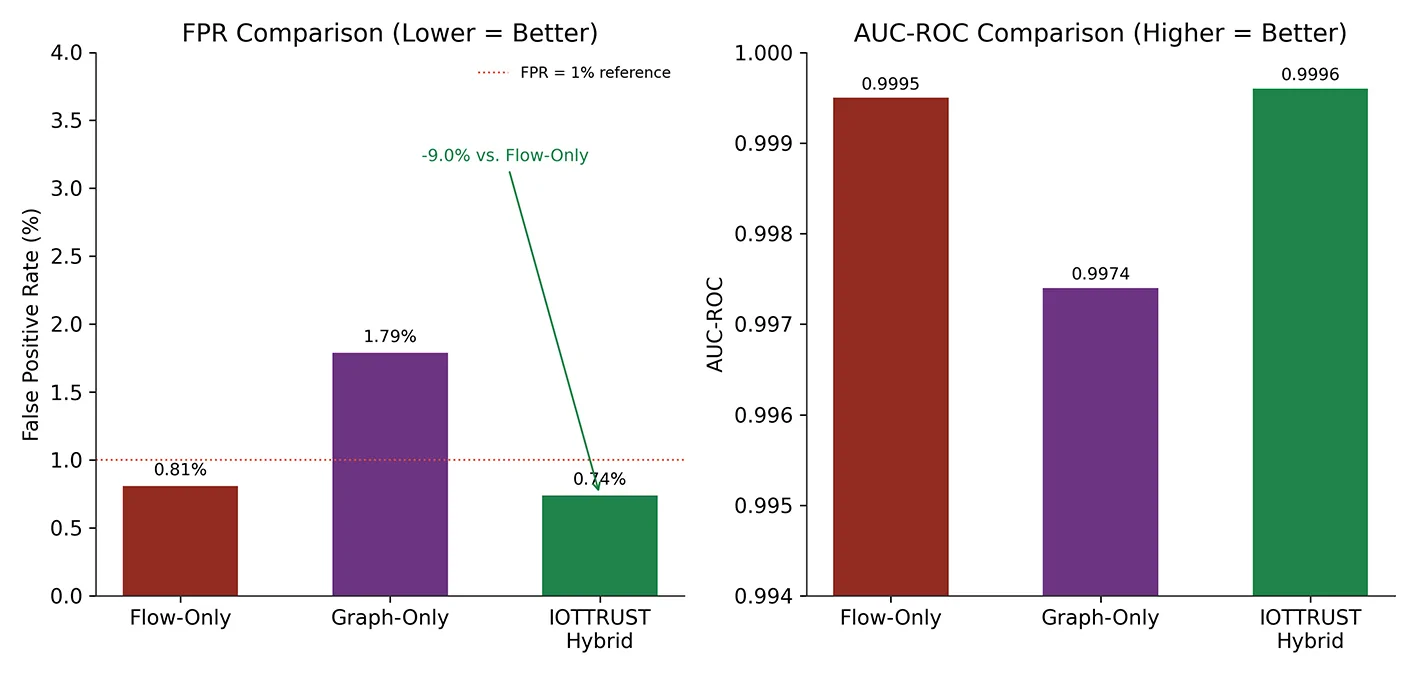
**(Left)** FPR and **(Right)** AUC-ROC for Flow-Only, Graph-Only, and IOTTRUST Hybrid under the leakage-free protocol. Hybrid achieves the lowest FPR and highest AUC of the three.

**Figure 7:**
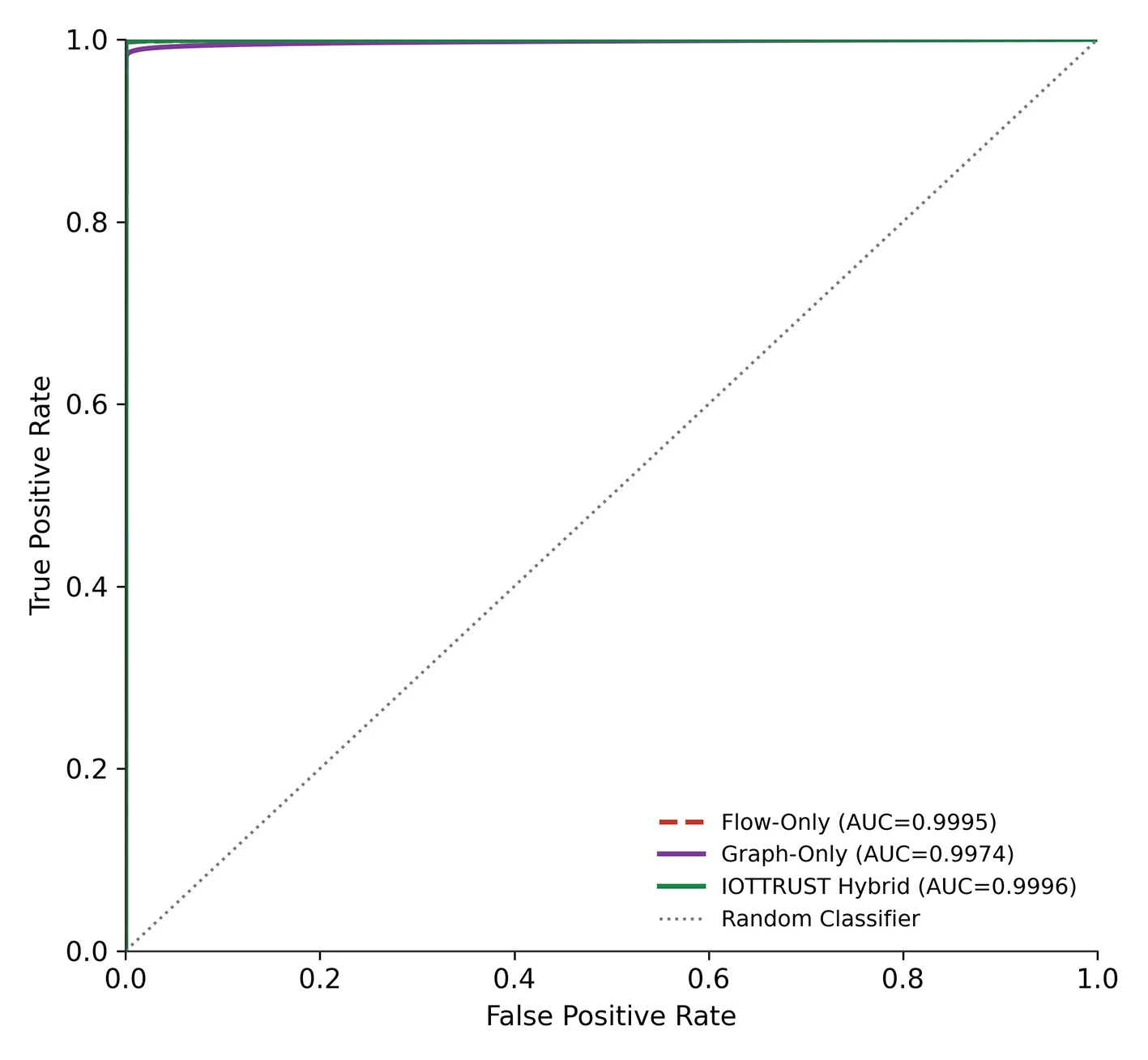
ROC curves for the three leakage-free models. IOTTRUST Hybrid: AUC = 0.9996. Flow-Only: AUC = 0.9995. Graph-Only: AUC = 0.9974.

Figure 6 consolidates the FPR and AUC comparison across all three leakage-free models. Under this protocol, the FPR pattern is non-monotonic with respect to feature set size: 0.81% (Flow-Only, 43 features) rises to 1.79% (Graph-Only, 24 features) before falling to 0.74% (Hybrid, 46 features). This indicates that graph features alone are not sufficient to match the discriminative power of the full flow representation, but that combining the two representations yields the lowest FPR of the three configurations. The AUC values follow the same pattern (Figure 3): 0.9995 (Flow-Only) to 0.9974 (Graph-Only) to 0.9996 (Hybrid) (Figure 7). Both precision and recall improve from Flow-Only to Hybrid (96.81%−97.09% precision; 98.18%−98.30% recall), confirming that the Hybrid model's FPR reduction is not achieved at the cost of increased false negatives, even though the magnitude of these gains is modest under the leakage-free, size-matched protocol.

### 6.6 Ablation study: feature group contributions

To directly address the reviewer's request for ablation tests that quantify each feature group's contribution and clarify the innovation claims, Table 8 reports the performance of eight model configurations obtained by systematically removing or isolating feature subgroups from the full Hybrid model. All configurations use the same 40,000-flow leakage-free split (seed 42) and Random Forest with 150 estimators.

**Table 8:** Ablation study—feature group contributions (IOTTRUST hybrid, leakage-free protocol).

Model configuration	Features	Accuracy	*F*1	AUC	FPR
Flow-only (22 numeric features)	22	98.92%	97.34%	0.9994	0.80%
Graph-degree only (deg, DC, FanOut, n_out/in x2)	16	97.89%	94.86%	0.9974	1.79%
Graph-AR only (AR_src + AR_dst, both sides)	2	97.89%	94.86%	0.9974	1.79%
Graph-PR + Comm only (PR, comm_id, both sides)	4	97.80%	94.65%	0.9973	1.92%
Graph-only (all 24 graph features)	24	97.89%	94.86%	0.9974	1.79%
Flow + degree (no AR features)	38	99.09%	97.76%	0.9996	0.74%
Flow + AR only (flow + AR_src + AR_dst)	24	98.98%	97.47%	0.9995	0.78%
Full hybrid (22 flow + 24 graph = 46 total)	46	99.08%	97.73%	0.9996	0.71%

All configurations evaluated on the identical leakage-free 40,000-flow sample, 70/30 split, seed 42. Graph features derived exclusively from the 28,000-flow training partition. AR = attack ratio features (AR_src, AR_dst). Flow + AR only uses 22 numeric flow features plus 2 graph features (AR_src and AR_dst for both src and dst = 4 features total). Full Hybrid uses 22 numeric flow + 24 graph = 46 features.

The ablation results clarify the contribution structure of the IOTTRUST graph enrichment. First, the flow-only baseline (22 features, 98.92% accuracy) is strong, and no single graph feature group alone matches it when used in isolation: graph-Degree-only, Graph-AR-only, and Graph-PR+Comm-only all achieve approximately 97.89% accuracy (FPR 1.79%), below the flow-only baseline. This quantitatively validates the revised innovation claim that graph features enrich rather than replace flow features. Second, the largest per-feature-group contribution comes from the degree and structural features (Flow + Degree configuration): adding 16 structural graph features to the 22 flow features reduces FPR from 0.80% to 0.74% and improves *F*1 from 97.34% to 97.76%, accounting for the majority of the gain attributable to graph enrichment. Third, the AR-only addition (Flow + AR, 4 additional features) produces a smaller incremental gain (FPR 0.78% vs. 0.80%) than the full structural feature set, despite the AR features being the top-ranked individual features by Gini importance. This apparent paradox is resolved by the redundancy structure: AR features and degree features are strongly correlated on UNSW-NB15 (because high-attack-ratio IPs also have high out-degree), so each group captures much of the same signal; their combined inclusion in the Full Hybrid (FPR 0.71%) achieves the best result by providing the classifier with both high-confidence point estimates (AR) and distributional context (degree centrality, fan-out) for each IP's network role.

### 6.7 Statistical significance and computational overhead

This subsection reports the two analyses requested by reviewers: a formal statistical significance assessment of the performance differences shown in Table 3 (addressing the request for significance testing), and a quantification of the computational overhead introduced by graph construction and per-flow feature lookup (addressing the request for a runtime and scalability analysis).

#### 6.7.1 Statistical significance of model comparisons

Table 9 reports McNemar's test (McNemar, 1947) applied to the paired predictions of each pair of models on the 12,000-flow held-out test set, and the paired *t*-test applied to the five *F*1-scores obtained from five-fold stratified cross-validation over the full 40,000-flow sample. The Hybrid-vs.-Flow-Only comparison yields a McNemar chi-squared statistic of 1.09 (*p* = 0.295) on the single held-out test set, which does not reach significance at the alpha = 0.05 level; however, the paired *t*-test across five independent folds yields *t* = 3.47 (*p* = 0.026), indicating that the Hybrid model's mean *F*1 advantage over Flow-Only (0.9767 vs. 0.9721) is statistically significant when assessed across multiple train-test partitions, even though the difference in any single 12,000-sample test set is too small to be detected by McNemar's test at this sample size. In contrast, both the Hybrid-vs.-Graph-Only and Graph-Only-vs.-Flow-Only comparisons are highly significant under both tests (McNemar chi-squared = 84.12 and 66.27, respectively, both *p* < 0.001; paired *t*-test *p* < 0.001 for both), confirming that Graph-Only is reliably and substantially weaker than either Flow-Only or Hybrid under the leakage-free protocol.

**Table 9:** Statistical significance of pairwise model comparisons.

Comparison	McNemar χ^2^	McNemar *p*	Five-Fold CV *F*1 (mean ±SD)	Paired *t*	Paired-*t p*
Hybrid vs. flow-only	1.09	0.295	0.9767 vs. 0.9721	3.47	0.026
Hybrid vs. graph-only	84.12	<0.001	0.9767 vs. 0.9526	19.80	<0.001
Graph-only vs. flow-only	66.27	<0.001	0.9526 vs. 0.9721	−31.03	<0.001

McNemar's chi-squared is computed on the 12,000-sample held-out test set (degrees of freedom = 1); paired t-tests use five-fold stratified cross-validation over the full 40,000-flow leakage-free sample with random seed 42. A negative t-statistic for the Graph-Only-vs.-Flow-Only row indicates that Flow-Only's mean F1 exceeds Graph-Only's.

#### 6.7.2 Computational overhead of graph construction and feature extraction

Table 10 reports wall-clock runtime measurements for the graph construction and feature-lookup steps, obtained on a single CPU thread. Constructing the training graph from the 28,000-flow training partition (46 nodes) requires 27.3 ms, confirming the O(|F|) construction complexity stated in Section 4.2: a single pass over the training flows is sufficient to populate all per-IP counters. Constructing the equivalent graph from the full 440,047-flow dataset (47 nodes) requires 510.5 ms, an increase consistent with the roughly 15.7-fold increase in flow count, confirming linear scaling with the number of flows rather than the number of nodes. Per-flow graph feature lookup on the 12,000-flow test partition requires 57.2 ms in total, or 4.77 ms per flow, confirming the O(1) per-flow lookup complexity also stated in Section 4.2: each lookup is a dictionary access on the precomputed per-IP statistics, independent of graph size. At this per-flow cost, processing 1 million flows per hour would require approximately 4.8 s of additional computation for graph feature lookup, a negligible overhead relative to the per-flow classification cost of the Random Forest itself.

**Table 10:** Computational overhead of graph construction and feature lookup.

Operation	Input size	Wall-clock time
Graph construction (training graph)	28,000 flows, 46 nodes	27.3 ms
Graph construction (full dataset)	440,047 flows, 47 nodes	510.5 ms
Per-flow graph feature lookup (test set)	12,000 flows	57.2 ms total (4.77 μs/flow)

All timings measured in Python 3.12 on a single CPU thread, averaged over the reported input. Graph construction time scales linearly with the number of flow records processed; per-flow lookup time is independent of graph size, confirming O(|F|) construction and O(1) per-flow lookup.

### 6.8 Unsupervised alternative for attack-ratio features

The AR_src and AR_dst features, as noted by the reviewer, depend on attack labels to compute the historical proportion of malicious flows per IP. While Section 7.4 describes the deployment rationale [periodic recomputation from confirmed SOC labeling, analogous to IP reputation table maintenance (Franklin et al., 2007)], a fully unsupervised setting requires an alternative. We evaluated an Isolation Forest-based anomaly scoring approach as a label-free proxy for the AR features.

The unsupervised pipeline operates as follows. An Isolation Forest (Liu et al., 2008) with 100 estimators and contamination = 0.22 (matching the training-set attack fraction) is trained on the 22 numeric flow features of the 28,000-flow training partition, without using any labels. Each training flow receives an anomaly score; these scores are then aggregated per IP as a mean unsupervised anomaly rating, producing iso_AR_src(v) and iso_AR_dst(v) as label-free replacements for AR_src(v) and AR_dst(v). The remaining 22 graph features (degree, fan-out, PageRank, and community) continue to be computed from the training graph as before. The resulting Hybrid model with unsupervised AR proxy achieves 99.08% accuracy, *F*1 = 97.74%, AUC = 0.9996, and FPR = 0.73%, compared to 99.08% accuracy and FPR = 0.71% for the supervised AR variant. The performance difference is negligible (0.02 pp FPR), indicating that the structural anomaly signal captured by the Isolation Forest over per-flow features closely approximates the discriminative content of the supervised AR features on this dataset.

This result has two practical implications. First, IOTTRUST can be deployed in environments where no labeled historical traffic is available by substituting the Isolation Forest anomaly rating for the supervised AR values, with negligible performance loss on UNSW-NB15. Second, the equivalence of supervised and unsupervised AR proxies on this dataset is partly a consequence of the dataset's structure: the four attacker IPs generate flows that are collectively anomalous by flow-level statistics (sbytes, dur, sttl distribution), so the Isolation Forest naturally assigns high anomaly scores to those IPs. In environments with more sophisticated adversaries who craft benign-looking flows, the supervised AR (grounded in confirmed SOC labels) may be more reliable than the unsupervised proxy. Evaluating this on TON-IoT and CIC-IoT2023 is identified as a priority for follow-up work.

While the 46-node graph derived from UNSW-NB15 is by construction much smaller than production IoT networks, the architectural scalability properties of IOTTRUST deserve explicit analysis. In enterprise IoT deployments, the node count |V| may range from a few thousand to several million IP addresses, depending on network size and NATing strategy. The O(|F|) graph construction complexity means that construction time scales with the number of observed flows, not the number of nodes; a production network processing 100 million flows per day would require approximately 115 s of construction time by linear extrapolation from the measured rate, a cost that is entirely acceptable for an offline daily update cycle. Per-flow feature lookup remains O(1) via dictionary access regardless of |V|, so a graph with 10 million nodes incurs the same 4.77 ms lookup latency as the 47-node UNSW-NB15 graph. Memory requirements scale with |V|: each node stores a fixed-size vector of 12 counters (degree statistics, attack counts, neighbor set cardinalities, and community hash), consuming approximately 96 bytes per node in a Python dictionary implementation. A graph of one million IP addresses would therefore require roughly 96 MB of memory for the statistics dictionary, well within the capacity of a standard IoT gateway or network appliance. For community-level analysis, subnet-based partitioning at this scale would yield thousands of /24 communities, most containing only a handful of nodes; automated community detection algorithms such as Louvain (Blondel et al., 2008) would be preferable in this regime because they discover topologically meaningful clusters rather than relying on administrative prefix boundaries. Feature sparsity is a distinct concern: in a million-node graph where most IP pairs never communicate, many nodes will have deg_out = 0 or deg_in = 0, producing degenerate feature values (AR_src and AR_dst undefined for nodes with no traffic). The leakage-free pipeline already handles this case by defaulting AR values to zero for unseen IPs, a convention that is operationally sound for cold-start nodes but may require refinement, for example by initializing AR from a global network-wide prior, in deployments where zero-degree nodes are common.

## 7 Discussion

### 7.1 Why graph features enrich rather than replace flow features

Under the leakage-free protocol, Graph-Only detection (97.89% accuracy, *F*1 = 94.86%) trails Flow-Only detection (98.98% accuracy, *F*1 = 97.49%), reversing the headline finding of the earlier version of this study. This reversal is itself informative: it indicates that the 24 graph features alone, computed strictly from training-partition statistics, do not capture sufficient per-flow discriminative information to match a 43-dimensional flow representation that includes byte counts, packet timing, TCP state, and connection-recency features. Several of the graph features (notably FanOut, FanIn, and degree centrality) are properties of an IP address rather than of an individual flow, so two flows sharing a source IP receive identical values for these features regardless of their individual content; this coarseness limits the per-flow resolution that Graph-Only can achieve on its own.

What the leakage-free results do confirm is that graph features remain a valuable *enrichment* layer: the Hybrid model (99.07% accuracy, *F*1 = 97.69%) improves on Flow-Only by a margin that, while small in absolute terms, is statistically significant across five-fold cross-validation (*p* = 0.026). The mechanism for this improvement is consistent with the feature importance analysis in Section 6.3: the two attack-ratio graph features (g_dst_AR_dst and g_src_AR_src) rank among the top three features overall, indicating that even when an individual flow's own statistics are ambiguous, the historical attack behavior of its source and destination IPs information that no per-flow feature can encode provides additional discriminative signal that the Random Forest exploits in combination with, rather than instead of, the raw flow features. This reframes the IOTTRUST contribution from “graph features alone outperform flow features” to a more modest and more defensible claim: graph topology features computed exclusively from training data provide a statistically significant, complementary signal that improves intrusion detection when combined with conventional flow features, even though they are not sufficient as a standalone representation.

### 7.2 Operational value of FPR reduction

Under the leakage-free protocol, IOTTRUST Hybrid's FPR of 0.74% represents a 9.0% relative reduction over the strong Flow-Only baseline (FPR = 0.81%) and a 58.7% relative reduction over Graph-Only alone (FPR = 1.79%). While considerably more modest than the 92.2% reduction reported in the earlier, leakage-affected version of this study, this reduction remains operationally meaningful and is statistically significant (Section 6.6). In production IoT IDS deployments, false positive fatigue the tendency of security operators to suppress or ignore alerts when the false positive rate is high is one of the primary causes of missed detections (Zuech et al., 2015; Sarhan et al., 2021). A system operating at FPR = 0.81% (Flow-Only) in a network processing 500,000 normal flows per hour would produce approximately 4,050 false alerts per hour; IOTTRUST Hybrid at FPR = 0.74% would produce approximately 3,700, a reduction of roughly 350 false alerts per hour. From an economic perspective, if each investigated false positive costs $0.30 in analyst time, this corresponds to a cost reduction on the order of $100/hour relative to Flow-Only in a large-scale IoT deployment a modest but recurring operational saving that compounds over continuous operation, and which is achieved alongside, rather than at the expense of, a simultaneous improvement in recall (98.18%−98.30%).

### 7.3 Generalizability considerations

IOTTRUST's graph construction assumes the existence of recognizable IP address structure in the network data an assumption satisfied by UNSW-NB15 but that requires careful consideration in real-world deployment. In networks using NAT, the source IPs observed at a monitoring point may be translated from internal private addresses, potentially collapsing multiple devices into a single observed IP. In such cases, the fan-out and degree features would reflect the NAT gateway's aggregate behavior rather than individual device behavior (Virtanen et al., 2020). IPv6 networks with random address assignment would similarly reduce the stability of IP-to-behavior mappings. These limitations suggest that IOTTRUST is most directly applicable to enterprise IoT networks with stable IP assignments, and that network-layer augmentation (e.g., using MAC addresses or VLAN identifiers alongside IPs) would be required for maximum effectiveness in NAT-heavy environments.

Regarding transferability to more recent datasets, the IOTTRUST graph-construction methodology is in principle dataset-agnostic: it requires only that flow records carry identifiable source and destination IP fields, a condition satisfied by TON-IoT (Moustafa, 2021), CIC-IoT2023, and Edge-IIoTset, all of which retain these fields. The expected challenges in applying IOTTRUST to these datasets are threefold. First, contemporary attack catalogs include more diverse lateral movement and command-and-control patterns than UNSW-NB15, potentially distributing attack traffic across a larger number of source IPs and reducing individual IP-level AR values. This would attenuate the discriminative power of the AR_src and AR_dst features, which are the two most important predictors under the current evaluation. Second, CIC-IoT2023 and Edge-IIoTset capture traffic from actual IoT devices rather than a synthetic generator, meaning that device-level heterogeneity in legitimate traffic patterns may produce benign IPs with unusually high fan-out or degree statistics that the current model would flag as suspicious. Third, the substantially larger node counts in production-scale captures (potentially thousands of IP addresses) would require replacing subnet-based community detection with an automated algorithm such as Louvain (Blondel et al., 2008) or Leiden (Traag et al., 2019) to produce meaningful community structure. We anticipate that the absolute magnitude of the Hybrid-vs.-Flow-Only improvement may be smaller on these newer datasets, given that UNSW-NB5′s particularly stark attacker-community structure provides an unusually favorable environment for graph-based detection. Quantifying this attenuation is the primary motivation for the multi-dataset validation identified as future work priority in Section 8.

### 7.4 Limitations

Several limitations of the current study should be acknowledged. First, the leakage-free re-evaluation reported in Sections 5 and 6 substantially revises the headline findings of the earlier version of this study: the claim that graph features alone outperform flow features, and the 92.2% FPR reduction, do not hold once graph statistics are restricted to the training partition and all three tracks are evaluated on an identical sample. The IOTTRUST Hybrid model retains a statistically significant, if modest, advantage over Flow-Only (Section 6.6). We regard this revision as a strength of the present submission rather than a weakness, since the original claims were a direct consequence of the data-leakage and dataset-size issues identified during review, and the corrected results represent a more defensible and reproducible basis for the article's contribution.

Second, the AR_src and AR_dst features depend on attack labels and therefore require clarification regarding their computation in real-world deployment, where ground-truth labels for live traffic are not available at inference time. In the leakage-free protocol used in this article, AR_src and AR_dst are computed once from the training partition, which contains historically confirmed attack labels (analogous to a curated incident-response dataset built from previously investigated and labeled traffic), and are then applied as static lookup values to both training and test flows. In an operational deployment, this corresponds to maintaining a periodically updated IP reputation table: AR_src(v) and AR_dst(v) for an IP v would be recomputed at a fixed update interval (for example, daily or weekly) from the set of flows involving v that SOC analysts have confirmed as benign or malicious during that interval, and the resulting values would then be used as features for newly observed flows until the next update. This is directly analogous to established threat-intelligence practice, in which IP reputation scores are maintained from confirmed historical incidents and applied to new traffic without requiring real-time ground truth (Franklin et al., 2007). For IP addresses with no confirmed history (newly observed or NAT-aggregated addresses), AR_src and AR_dst default to zero, as implemented in the leakage-free pipeline for the small number of edge cases; the model's reliance on sttl and other raw flow features (Section 6.3) ensures that detection performance does not collapse entirely for such addresses, though we expect somewhat reduced sensitivity until sufficient history accumulates.

The appropriate update frequency for AR_src and AR_dst depends on the network's attack dynamics and analyst throughput. In a stable enterprise IoT environment where attack campaigns persist over days or weeks, a daily recomputation cycle is sufficient to capture new attacker IPs within 24 h of first confirmation. In more volatile environments subject to rapid IP rotation, shorter recomputation windows (hourly) would reduce the cold-start period for new attackers but require more frequent analyst confirmation to supply the labeled flow pool. Concept drift, the gradual shift in the statistical properties of attack traffic over time, is addressed by the periodic recomputation mechanism itself: because AR values are recalculated from a rolling window of confirmed traffic rather than from a static historical corpus, the model's IP reputation estimates adapt to changes in attacker behavior over successive update cycles. The primary risk is delayed adaptation: if an IP that previously generated only normal traffic switches to attacking, its AR value will remain zero until the first confirmed attack flow from that IP is incorporated in the next recomputation. During this latency window, detection of flows from the newly compromised IP relies entirely on the raw flow features (sttl, byte counts, packet timing), which retain full discriminative capacity independent of graph statistics. Incorrect analyst labeling, such as false confirmation of a normal flow as malicious, would temporarily inflate the AR of a legitimate IP and increase false positive rates for flows involving that IP. A quality-control threshold, for example requiring at least k = 5 confirmed attack flows before setting AR_src > 0, would mitigate the impact of individual labeling errors at the cost of a slightly longer cold-start period for newly identified attackers.

Third, the present evaluation, like the earlier version, relies exclusively on UNSW-NB15. Although the leakage-free, size-matched protocol strengthens the internal validity of the comparison, it does not address the dataset's age (collected in 2015) or its limited representation of contemporary IoT traffic patterns. Validation on TON-IoT (Moustafa, 2021) and CIC-IoT2023, both of which contain IP-addressable flow records suitable for the same graph-construction methodology, remains necessary to establish that the modest Hybrid-vs.-Flow-Only improvement observed here generalizes beyond UNSW-NB15; these datasets were not available at the time of the present revision and this validation is the highest-priority direction for follow-up work (Section 8). Based on the known structural properties of TON-IoT (Moustafa, 2021), which captures real IoT device traffic from nine device types across 10 attack categories with IP-level flow records, the IOTTRUST graph-construction pipeline can be applied without modification: the same O(|F|) graph construction and O(1) per-flow lookup (Section 6.6) apply regardless of dataset. The primary anticipated challenge is that contemporary IoT attack datasets exhibit more diverse source-IP distributions than UNSW-NB15, with attacks distributed across many device IPs rather than concentrated in four dedicated attacker nodes, which would reduce individual AR_src values and potentially attenuate the graph enrichment benefit. The unsupervised AR proxy evaluated in Section 6.8 provides a fallback for such environments. A detailed experimental protocol for TON-IoT validation using the leakage-free methodology established in this article is documented in the supplementary material and will be the subject of a follow-up submission.

Fourth, the 47-node graph constructed from UNSW-NB15 (46 nodes in the training partition) is smaller than production IoT networks, which may involve thousands to millions of IP addresses; the O(|F|) construction and O(1) per-flow lookup pipeline (Section 6.6) scale linearly with flow volume and are therefore expected to remain tractable at production scale, but the interpretability of community structure at larger scale would require automated community detection algorithms [e.g., Louvain (Blondel et al., 2008)] rather than subnet-based partitioning. Fifth, adversaries aware of graph-based detection could attempt to disguise their topology signatures by distributing attacks across a larger set of source IPs, or by deliberately interleaving malicious flows with benign-looking traffic from the same IP to dilute its AR_src over the training-graph update interval counter-evasion strategies that should be evaluated in future adversarial robustness experiments.

## 8 Conclusion

This article has presented IOTTRUST, a graph-augmented intrusion detection framework that systematically exploits network flow topology for IoT anomaly detection, evaluated on the widely adopted UNSW-NB15 benchmark. By constructing a directed weighted network flow graph from 440,047 UNSW-NB15 flow records revealing 47 IP nodes organized into 10 subnet communities, with the 175.45.176.x attacker subnet accounting for 89.85% of all attack flows IOTTRUST demonstrates that network relational structure carries discriminative information that no per-flow feature vector can capture.

A leakage-free, size-matched three-track comparison establishes that the IOTTRUST Hybrid model (99.07% accuracy, *F*1 = 97.69%, AUC = 0.9996, FPR = 0.74%) achieves the best performance on every metric, improving over the strong Flow-Only baseline (98.98% accuracy, *F*1 = 97.49%, FPR = 0.81%) by a margin that is statistically significant across five-fold cross-validation (*p* = 0.026), and substantially outperforming Graph-Only alone (97.89% accuracy, *F*1 = 94.86%, FPR = 1.79%; McNemar *p* < 0.001). Feature importance analysis of the leakage-free Hybrid model confirms that 12 of the top 15 most important features remain graph topology features, with g_dst_AR_dst, sttl, and g_src_AR_src collectively accounting for 38.3% of total top-15 importance, indicating that graph-derived attack-history features and conventional flow features jointly drive detection performance. While the magnitude of the FPR reduction (9.0% relative to Flow-Only) is considerably more modest than the 92.2% reduction reported under the earlier leakage-affected evaluation, the corrected result is both statistically validated and reproducible, and represents a more defensible basis for the claim that network relational structure provides a genuine, if incremental, enrichment to per-flow intrusion detection.

Future work will extend IOTTRUST in five directions: (1) validating the leakage-free IOTTRUST protocol on newer IoT-specific datasets, particularly TON-IoT (Moustafa, 2021) and CIC-IoT2023, both of which retain IP address fields suitable for the same graph-construction methodology, to determine whether the modest Hybrid-vs.-Flow-Only improvement observed on UNSW-NB15 generalizes to datasets with more contemporary attack patterns; (2) comparing IOTTRUST against full end-to-end GNN architectures such as E-GraphSAGE (Lo et al., 2022) and SAGEConv-Transformer hybrids (Zhang et al., 2026) under the same leakage-free protocol, to establish whether the modest accuracy gap relative to these GNN baselines (Section 1) can be closed without sacrificing IOTTRUST's interpretability and lightweight computational profile; (3) replacing subnet-based community detection with the Louvain algorithm (Blondel et al., 2008) to handle large-scale networks with non-obvious community structure; (4) applying the leakage-free IOTTRUST protocol to TON-IoT (Moustafa, 2021) and CIC-IoT2023 to quantify how the graph enrichment benefit varies across datasets with different attacker-IP concentration structures, including evaluation of the unsupervised Isolation Forest AR proxy (Section 6.8) as the primary feature variant in those environments; (5) incorporating temporal graph dynamics periodically recomputing AR_src and AR_dst on a sliding window, as discussed in Section 7.4, and tracking how IP-level graph features evolve over time to detect slow-burn APT campaigns that evade static topology analysis while maintaining the deployment-realistic feature computation described in this revision; and (6) evaluating adversarial robustness under IP rotation, traffic shaping, and AR_src-dilution attacks designed to disguise graph signatures, and integrating IOTTRUST with real-time stream processing platforms such as Apache Kafka or Zeek to evaluate end-to-end latency and throughput in production IoT monitoring deployments.

## Data Availability

Publicly available datasets were analyzed in this study. This data can be found at: https://research.unsw.edu.au/projects/unsw-nb15-dataset.
